# Supplementary material for: Betulinic Acid-Mediated Tuning of PERK/CHOP Signaling by Sp1 Inhibition as a Novel Therapeutic Strategy for Glioblastoma
Source: Cancers (Basel). 2020 Apr 15;12(4):981. doi: 10.3390/cancers12040981 (PMC7226172; doi:10.3390/cancers12040981)
Supplement: Supplementary file 1 [file cancers-12-00981-s001.pdf]

Supplementary Materials

Betulinic Acid-Mediated Tuning of PERK/CHOP Signaling by Sp1 Inhibition as a Novel Therapeutic Strategy for Glioblastoma

Wei-Lun Lo, Tsung-I Hsu, Wen-Bin Yang, Tzu-Jen Kao, Ming-Hsiao Wu, Yung-Ning Huang, Shiu-Hwa Yeh and Jian-Ying Chuang

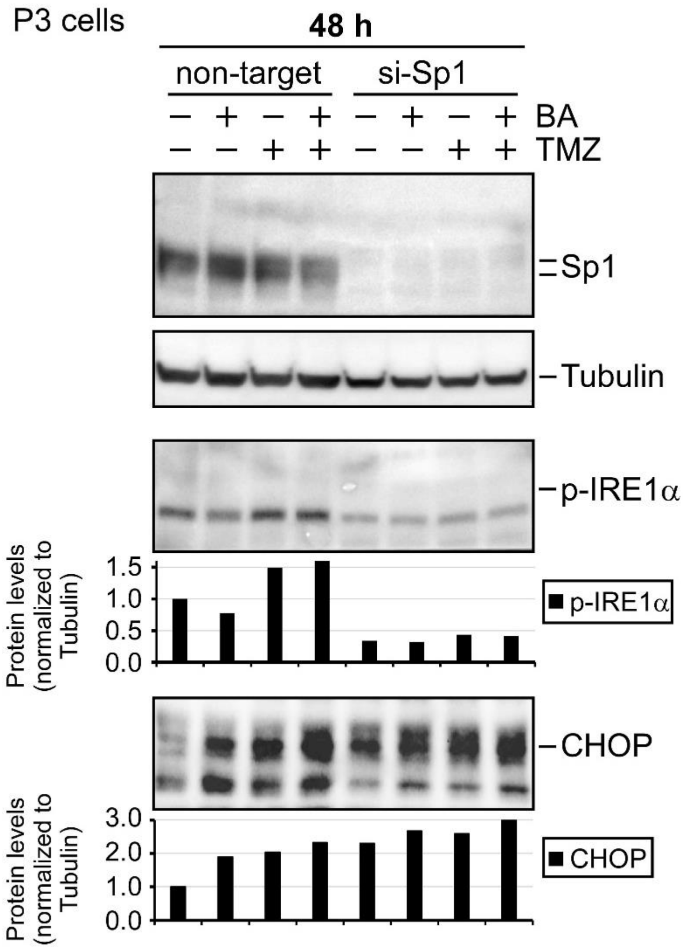

Figure S1. Roles of Sp1 in regulating UPR signaling. P3 cells were transfected with a Sp1-specific siRNA or a control non-targeting siRNA as indicated. After 1 day of transfection, cells were treated with BA (20 μM) and/or TMZ (100 μM) for 2 days. Cellular lysates were then collected and analyzed by western blotting with the indicated antibodies.

Fig.3A and 3B

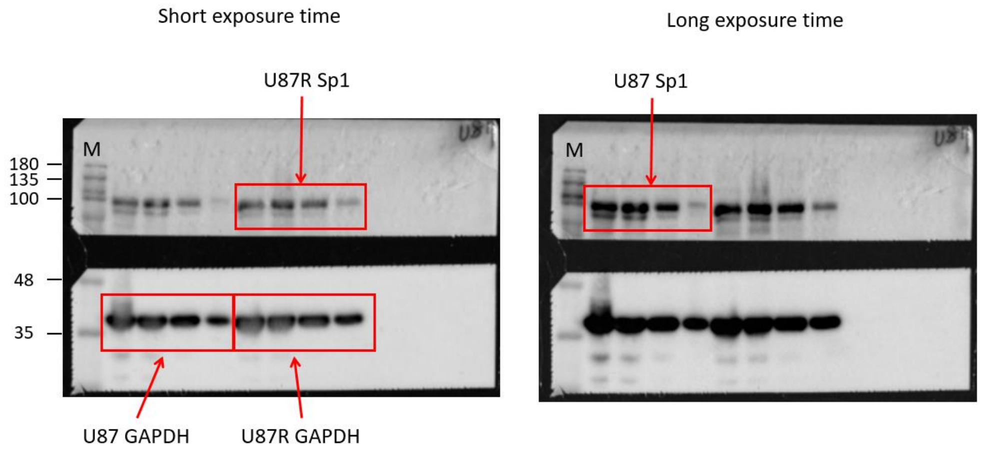

Fig.3A and 3B

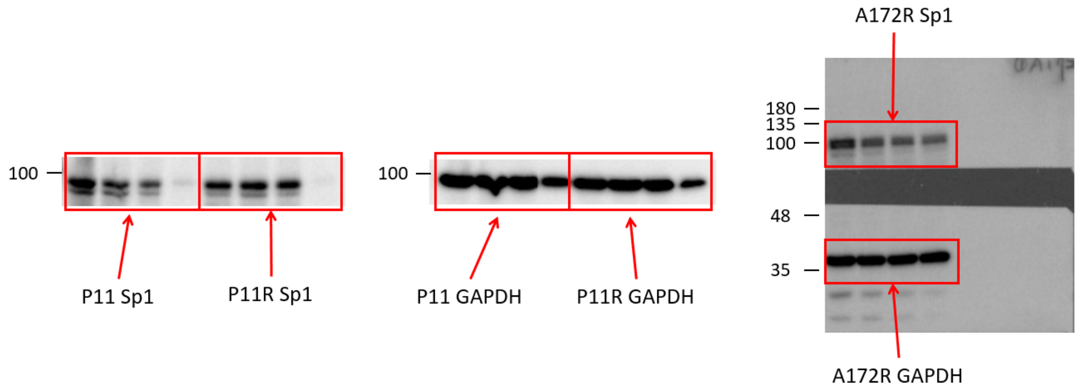

Fig.3C

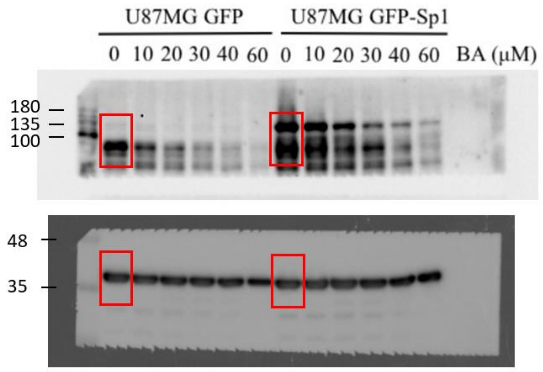

Fig. 4D

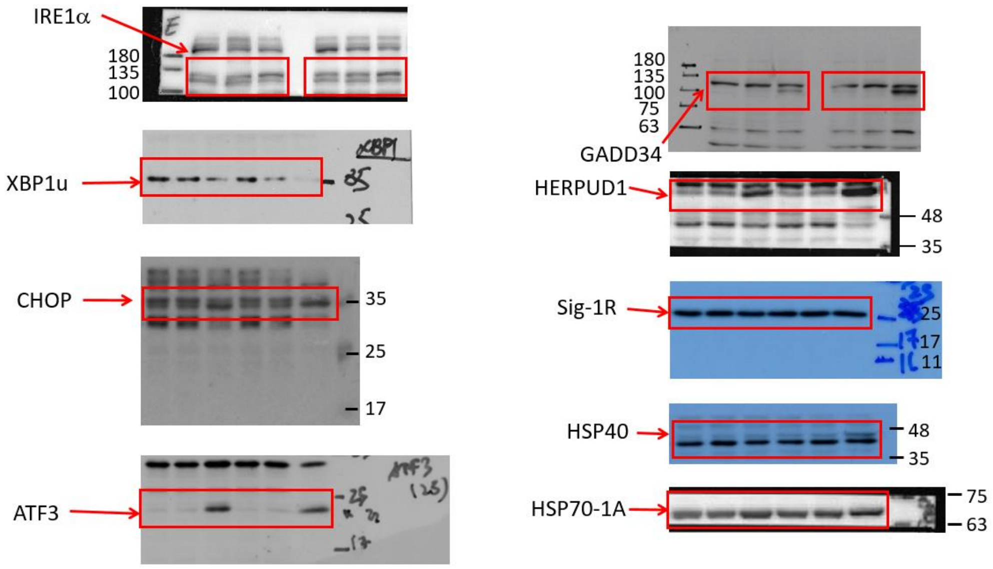

Fig. 4D

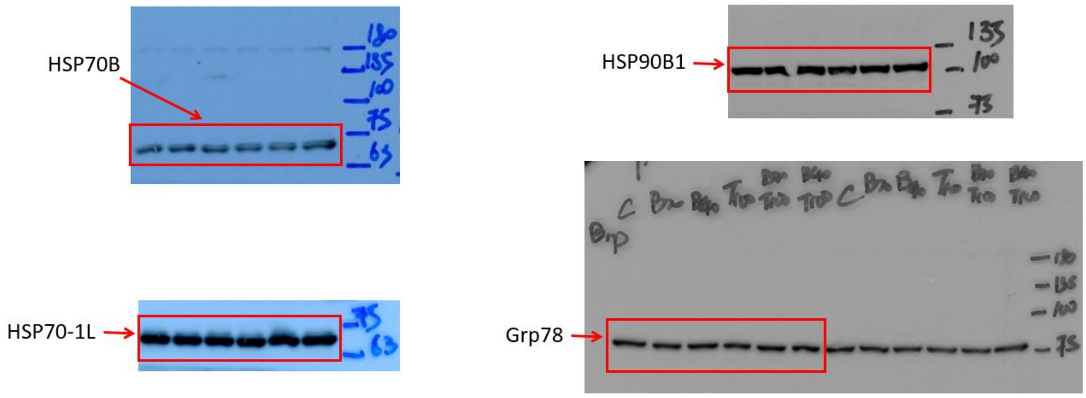

Fig. 5

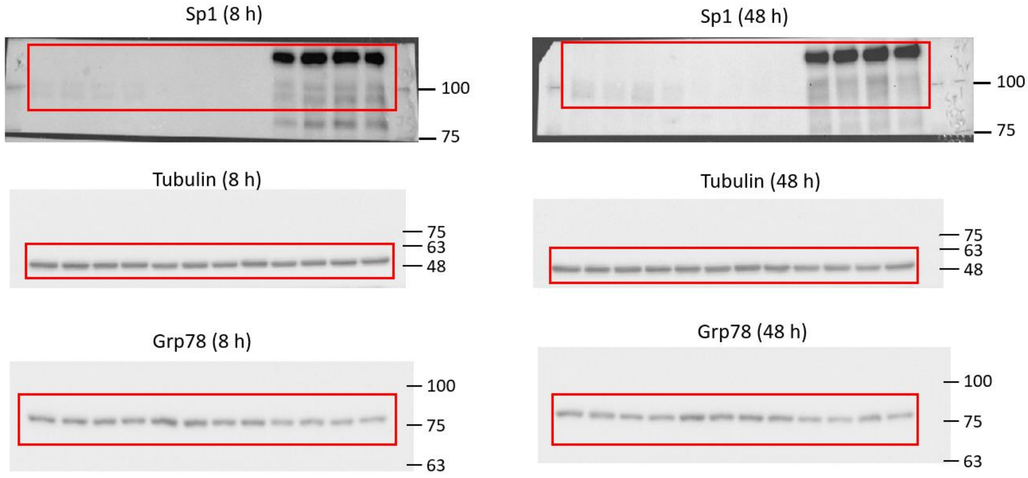

Fig. 5

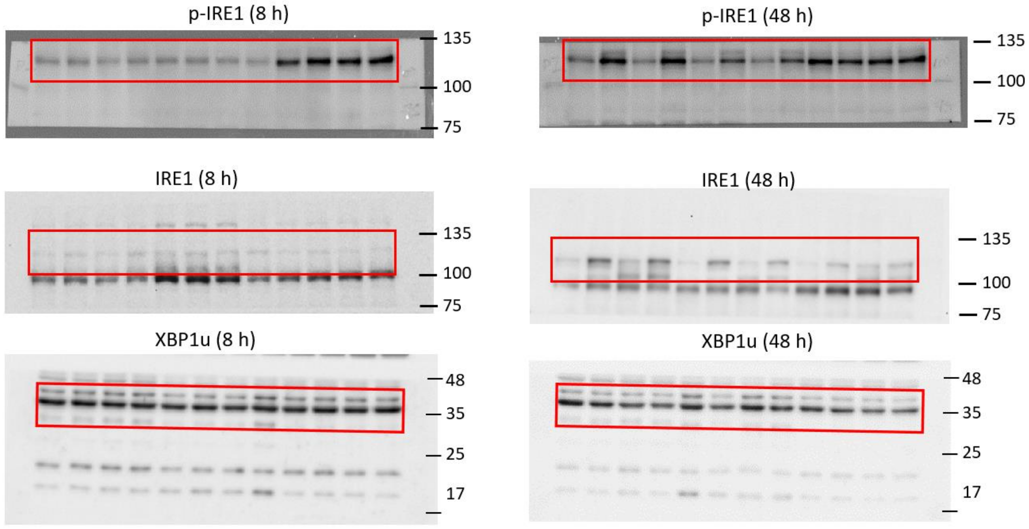

Fig. 5

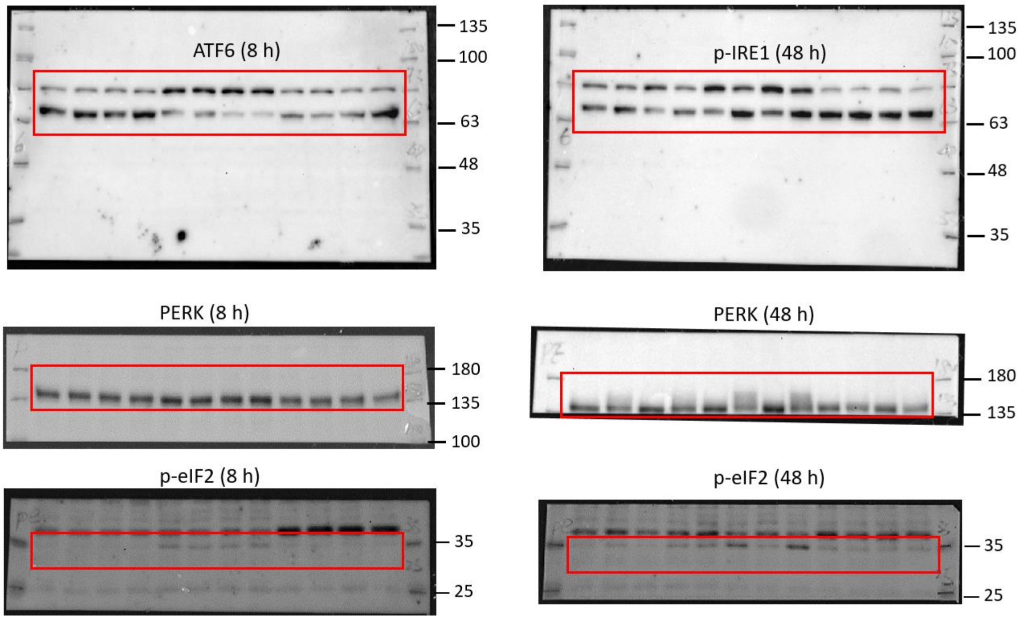

Fig. 5

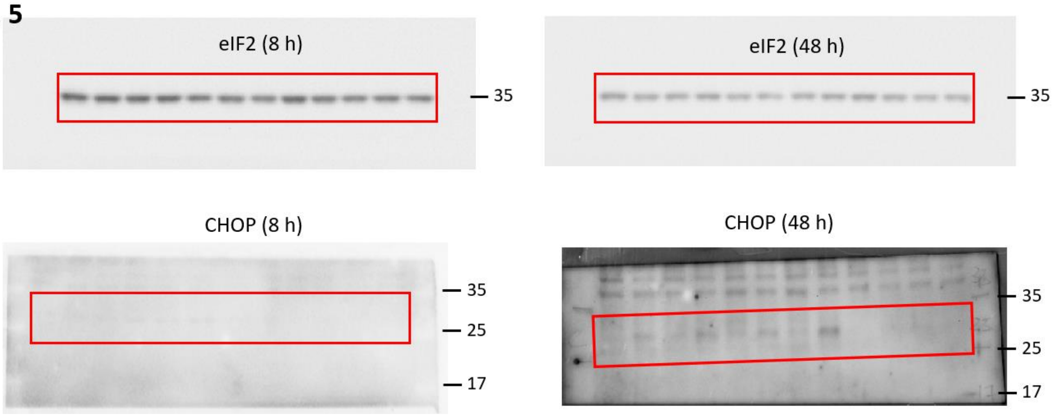

Fig. 6B

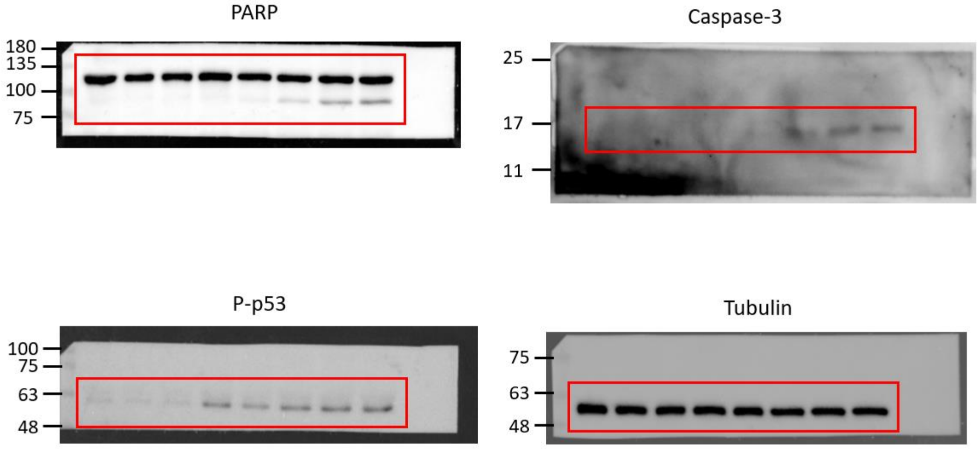

Fig. 6D

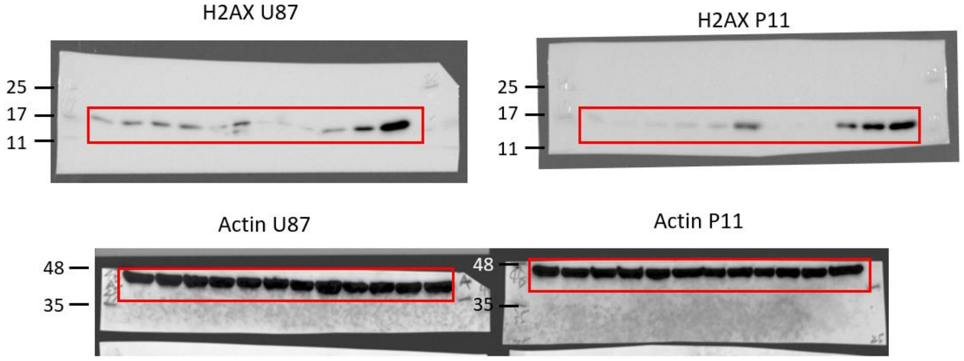

Fig. 6E

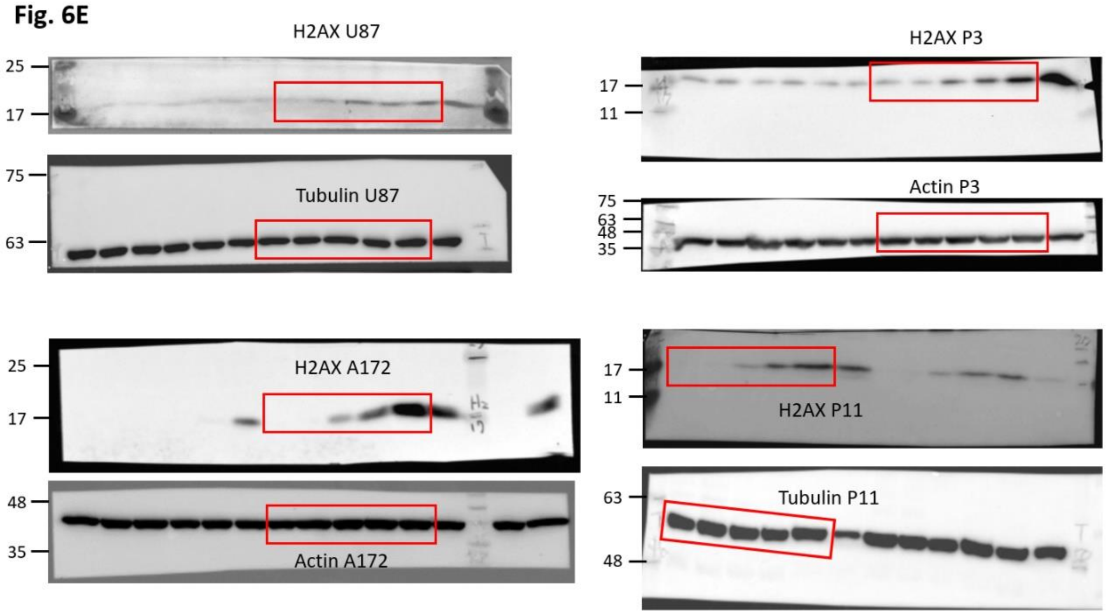

Western Blot.

© 2020 by the authors. Licensee MDPI, Basel, Switzerland. This article is an open access article distributed under the terms and conditions of the Creative Commons Attribution (CC BY) license (http://creativecommons.org/licenses/by/4.0/).
